# Effectiveness of the INSIGHT Program with Perpetrators of Sexual Violence Against Girls: A Non-RCT Pilot Study

**DOI:** 10.3390/bs16060991

**Published:** 2026-06-15

**Authors:** Marta Sousa, Olga Cunha, Rui Abrunhosa Gonçalves, Andreia de Castro-Rodrigues

**Affiliations:** 1 HEI-Lab—Digital Human-Environment Interaction Labs, Lusófona University, Porto University Center, Faculty of Psychology and Education, Rua Augusto Rosa, No. 24, 4000-098 Porto, Portugal; 2 Psychology Research Center (CIPSI), School of Psychology, University of Minho, 4710-057 Braga, Portugal; olga.cunha@psi.uminho.pt; 3 CINTESIS.UPT Research Centre, Portucalense University, R. Dr. António Bernardino de Almeida 541, 4200-072 Porto, Portugal; rui.goncalves@upt.pt; 4 William James Center for Research, ISPA—Instituto Universitário de Ciências Psicológicas, Sociais e da Vida, 1149-041 Lisboa, Portugal; arodrigues@ispa.pt

**Keywords:** sexual violence, INSIGHT program, child sexual abuse, reliable change index

## Abstract

Sexual violence against young girls is a complex phenomenon encompassing multiple forms of abuse and leading to numerous negative outcomes. Therefore, rehabilitation measures play a critical role in reducing recidivism and enhancing victims’ safety. This study examined preliminary results of a 25-session individual intervention program (the INSIGHT Program) designed for individuals who sexually offended in Portugal, in both prison and community settings. In this pilot clinical trial, 19 participants were assigned to one of two conditions: INSIGHT plus treatment as usual (TAU) or TAU alone. Data was collected at baseline and at the end of the intervention. Proximal outcomes (e.g., attitudes toward child sexual abuse, victim empathy, interpersonal problems, and early maladaptive schemas) were assessed. The Reliable Change Index (RCI) was computed. Results indicated that participants receiving INSIGHT plus TAU evidenced greater clinical improvements in empathy toward victims and interpersonal problems compared with participants receiving TAU alone. Overall, the intervention demonstrates potential to reduce some risk factors among individuals who perpetrated sexual violence against young girls. However, we highlight the need to reconsider the work carried out on EMSs, particularly regarding the techniques used.

## 1. Introduction

Child Sexual Abuse (CSA) is a serious public health issue and a violation of human rights, with negative, severe and lasting consequences for victims, their families, and society (Hailes et al., 2019). The literature indicates high prevalence rates of CSA perpetration, which vary considerably depending on whether they are assessed through self-report or official records (Stoltenborgh et al., 2011). Challenges in defining prevalence data stem from difficulties in disclosure and reporting (Wallis & Woodworth, 2020), often due to fear, shame, and sometimes a lack of support and understanding (Lemaigre et al., 2017). Nevertheless, a global meta-analysis found that 8–31% of girls and 3–17% of boys experience CSA before the age of 18 (Barth et al., 2013). More recent meta-analyses have reported similarly high rates (22.1–24% for females and 9.2% for males, (Pan et al., 2021)). In Portugal, the most recent available data indicate that 1041 CSA crimes were recorded in 2024 (Direção Geral de Reinserção e Serviços Prisionais, 2024), an increase compared to the previous year, with half of the cases being girls (53.4%). According to the official data from Portugal’s Annual Internal Security Report, the crimes that recorded the highest number of investigations initiated and arrests in 2025 were CSA offenses. These crimes were predominantly perpetrated by men (90.6%), mostly within a family context (49.5%), particularly against victims aged between 8 and 13 years (Sistema de Segurança Interna, 2026).

Given the high prevalence and serious consequences of this phenomenon, establishing effective public policies to address it is imperative. Developing interventions specifically targeting men convicted of CSA is crucial for reducing the risk of reoffending (Barros et al., 2022; Gannon et al., 2019; Marshall, 2020). However, worldwide efforts have often focused on measures that, although well-intentioned, have shown little evidence of reducing recidivism (Zgoba & Mitchell, 2023), and in some cases may even produce harmful effects. These approaches primarily emphasize control and monitoring rather than rehabilitation, such as sex offender registries, community notification, and residence restrictions (Zgoba & Mitchell, 2023). In contrast, psychological intervention represents a rehabilitative strategy that is essential not only for these men but also for potential future victims and society (Marshall, 2020). Effective psychological treatment can contribute to reducing the number of victims, mitigating the harmful impact of these offenses, and lowering associated financial costs (Marshall, 2020).

Psychological interventions for individuals who have sexually offended against adults and children have been shown to reduce sexual recidivism rates (Barros et al., 2022; Gannon et al., 2019). However, recent findings regarding the effectiveness of programs specifically targeting individuals who perpetrated CSA remain discouraging (Grønnerød et al., 2015). Limitations in study quality and intervention design have hindered the accumulation of robust evidence on treatment effectiveness (Grønnerød et al., 2015). Nonetheless, the recent literature outlines several key considerations for practitioners when developing intervention programs (Grønnerød et al., 2015).

Researchers have criticized intervention programs for focusing primarily on individuals’ deficits while overlooking the underlying cognitive structures that drive behavior (Brazão et al., 2018; Keulen-de Vos et al., 2022). Information processing operates at multiple levels—schemas, processes, and cognitive products (Hollon & Kriss, 1984; Segal & Shaw, 1988). Schemas are core cognitive structures that organize perceptions of the self, others, and the world (Beck, 1963), shaping attention, interpretation, and belief formation. Therefore, targeting surface-level cognitions alone is insufficient; effective interventions must also address deeper schemas and distorted self- and other-related beliefs that increase vulnerability to offending (Gannon, 2016). Therefore, Schema Therapy (ST) (Young et al., 2003) has been proposed for forensic populations, including individuals who perpetrated CSA, to better address the developmental and cognitive processes underlying sexual offending (Turhan et al., 2024). ST focuses on early maladaptive schemas (EMSs)—dysfunctional, self- and relationship-related patterns rooted in adverse childhood experiences and temperament (Young et al., 2003). When activated, EMSs bias information processing and contribute to persistent cognitive distortions and maladaptive behaviors (Brazão et al., 2018). Research has identified several EMSs commonly associated with individuals who perpetrate CSA, such as abandonment, distrust/abuse, emotional deprivation, defectiveness/shame, social isolation, subjugation, and emotional inhibition (Brazão et al., 2018; Turhan et al., 2024). These schemas are linked to insecure attachment styles and relational difficulties, which may intensify negative affect, isolation, and deviant sexual fantasies (Chakhssi et al., 2013; Turhan et al., 2024). Addressing these deeper psychological structures may therefore promote more enduring cognitive and behavioral change (Brazão et al., 2018).

A growing body of evidence supports the use of ST in both forensic and non-forensic populations (Turhan et al., 2024; Sousa et al., 2024). A recent systematic review of 11 studies found ST—alone or combined with other approaches—to be a promising intervention for individuals who commit various offenses (Sousa et al., 2024). However, its specific application to individuals who have perpetrated CSA remains limited. In the Portuguese context, to the best of our knowledge, there is only one intervention program available for adult individuals convicted of sexual offenses, regardless of whether the crimes were committed against adults or children. This program follows a cognitive-behavioral approach and does not specifically target EMSs or address the distinct needs of individuals who commit sexual offenses against children (Direção Geral de Reinserção e Serviços Prisionais, 2026). Given the mixed evidence regarding the effectiveness of interventions for men who have perpetrated CSA, and the literature highlighting EMSs as key treatment targets due to their association with risk factors for sexual recidivism, the present study aims to determine the short-term effects of the INSIGHT Program—an individual intervention grounded in ST (with further details provided in Section 2)—on factors associated with sexual recidivism in a sample of Portuguese CSA perpetrators serving prison and community sentences, using a non-randomized controlled trial design (non-RCT).

## 2. Materials and Methods

### 2.1. Participants

Participants were selected based on the following exclusion criteria: (1) the presence of cognitive impairments, psychotic symptoms, or pronounced psychopathy traits; (2) prior completion of an intervention program targeting sexual offenses; (3) convictions for child pornography and/or sexual crimes against adults; (4) a remaining prison sentence of less than 12 months at the start of the program; and (5) being female. The sample consisted of 19 men aged between 25 and 61 (M = 45.11; SD = 9.04), assigned to two conditions: a treatment condition (*n* = 10) and treatment-as-usual (TAU) condition (*n* = 9). Participants mostly had attained a 9th-grade education/middle school (*n* = 9; 47.4%) or a 6th-grade of education (*n* = 4; 21.1%) (see Table 1).

### 2.2. Instruments

#### 2.2.1. Young Schema Questionnaire (YSQ-S3)

The YSQ—S3 (Young, 2005; Rijo, 2009) is a 90-item self-report questionnaire. It assesses the 18 EMSs proposed by Young et al. (2003) through a 6-point Likert-type scale (1 = completely untrue about me; 6 = describes me perfectly). Studies with Portuguese samples have demonstrated a stable 18-factor structure with moderate item–total correlations and high internal consistency (α = 0.97) (Rijo, 2009). In the INSIGHT Program, seven schemas were incorporated as underlying relationship difficulties: emotional deprivation (5 items; α = 0.82), social isolation/alienation (5 items; α = 0.81), emotional inhibition (5 items; α = 0.81), defectiveness/shame (5 items; α = 0.86), mistrust/abuse (5 items; α = 0.78), abandonment/instability (4 items; α = 0.81), and subjugation (5 items; α = 0.78).

#### 2.2.2. Hanson Sex Attitude Questionnaire (HSAQ)

The Portuguese adaptation of the HSAQ (Hanson et al., 1994; Sousa et al., 2025c) is a 9-item self-report instrument intended to assess cognitive distortions related to CSA and sexuality. An exploratory factor analysis revealed two distinct factors: Misconceptions about CSA (HSAQ F1; 4 items) and Sexual Drive/Preoccupation (HSAQ F2; 5 items). Responses are rated on a 5-point ordinal scale ranging from 1 (strongly disagree) to 5 (strongly agree), with higher scores indicating greater endorsement of cognitive distortions. The internal consistency of the Portuguese version ranged from moderate (α = 0.67) to good (α = 0.74).

#### 2.2.3. Victim Empathy Distortion Scale (VES)

VES (Beckett & Fisher, 1994; Sousa et al., 2025a) is a 28-item self-report measure developed to evaluate individuals’ awareness of the consequences of their offenses on victims. In its Portuguese adaptation, the instrument is structured into two dimensions: positive misattributions of pleasure in sexual abuse (VES F1; 18 items) and negative attributions regarding CSA (VES F2; 10 items). Higher scores on the first dimension reflect stronger cognitive distortions about CSA, whereas higher scores on the second dimension indicate greater recognition of the harmful impact of abusive experiences on victims. Items are rated on a 5-point Likert scale from 0 (strongly disagree) to 4 (strongly agree). The scale shows solid psychometric qualities, with Cronbach’s alpha values of 0.94 for the first factor and 0.87 for the second factor (Sousa et al., 2025a).

#### 2.2.4. Inventory of Interpersonal Problems

The Inventory of Interpersonal Problems (IIP-32) (Barkham et al., 1996; Faustino & Vasco, 2020) is a self-report measure designed to assess difficulties in interpersonal functioning across eight domains. These include domineering/controlling (4 items), referring to a tendency to exert excessive control or manipulation over others; intrusive/needy (4 items), reflecting difficulties in respecting others’ boundaries and an excessive focus on one’s own needs; self-sacrificing (4 items), characterized by excessive generosity, care, trust, and permissiveness; overly accommodating (4 items), involving a tendency to be easily exploited or taken advantage of; nonassertive (4 items), referring to difficulties in expressing one’s own needs; socially avoidant (4 items), characterized by anxiety and avoidance in social interactions; cold/distant (4 items), reflecting limited emotional closeness and affection toward others; and vindictive/self-centered (4 items), involving hostile, egocentric, or resentful interpersonal patterns. The instrument comprises 32 items, answered on a 5-point Likert scale ranging from 0 (not at all) to 4 (extremely). In the Portuguese validation study, internal consistency values ranged from moderate to high, with Cronbach’s alpha coefficients between 0.63 and 0.89 (Faustino & Vasco, 2020).

### 2.3. Interventions

#### 2.3.1. INSIGHT Plus Treatment as Usual

The INSIGHT Program was developed in 2022 for males who have perpetrated CSA. It follows a multilevel, multimodal approach, comprising (1) an initial individual Motivational Interviewing (MI) session to enhance treatment adherence, with the number of sessions in this phase varying according to each patient’s motivational stage, and (2) a structured individual intervention program with 25 sessions. Grounded in ST (Young et al., 2003), the core program includes weekly 60 min sessions. The first phase focuses on strengthening self-esteem and self-efficacy, emotional regulation, cognitive restructuring, communication skills, and EMSs. The second phase is individualized and targets participants’ offense cycles, including risk factors, cognitive distortions, and underlying emotional states, with a strong emphasis on developing victim empathy. The program aims to reduce the impact of EMSs on social information processing and to promote adaptive emotional regulation and prosocial beliefs, thereby enhancing psychological well-being. Participants in the treatment group also received TAU provided by Portuguese prison and probation services, including sentence supervision, monitoring of educational and occupational activities, and psychological and/or psychiatric support upon request.

#### 2.3.2. Treatment as Usual

Participants assigned to the control group received TAU within Portuguese prison and probation settings. This typically involved monitoring of educational attendance, occupational and work-related activities, supervision of the individual sentence plan, and access to psychological and/or psychiatric support upon request. They did not participate in the INSIGHT sessions during the study period. Additionally, some individuals were offered the possibility of attending a prison-based group intervention program.

### 2.4. Procedure

In the initial phase, the study was submitted to and approved by the Ethics Committee of the University where the research was conducted. It was also approved by the General Directorate of Reintegration and Prison Services of the Portuguese Ministry of Justice as well as by the Psychology Association of the University of Minho. Subsequently, the entities involved in data collection (i.e., two prisons in Portugal and one community-based service) were contacted to assist in identifying potential participants with these convictions. Potential participants were then approached and informed about the objectives and conditions of participation and were also provided with information regarding the intervention program. It was clearly explained that participation was voluntary and that refusal to participate would not have any implications for their legal proceedings. Those who agreed to participate signed an informed consent form and completed a psychological assessment (i.e., the scales mentioned in Section 2.2), after which they were assigned to conditions according to availability to benefit at the time of the intervention. Psychological assessment of participants in the treatment condition took place at two time points, individually and in a private room, through the completion of paper-and-pencil questionnaires. These two data collection time points were: one week prior to the start of the program (pre-assessment) and one week after completing the program (post-test assessment). Participants in the control condition were assessed at the same time points. Each participant was assigned a specific code to facilitate the linkage of data across assessment waves. The intervention program was delivered by a psychologist with training and experience in psychological interventions with individuals convicted of CSA. Treatment integrity was ensured through regular supervision meetings with the research team.

### 2.5. Data Analysis

Quantitative analyses were conducted using non-parametric procedures, given the small sample size. Baseline comparisons of the outcome variables between groups employed Mann–Whitney tests. Between-group comparisons post-test were also examined with Mann–Whitney U tests, whereas changes within each group across time were assessed using Wilcoxon signed-rank tests conducted in SPSS (version 29). Given the pilot design and the small sample size, intervention effects were evaluated based on both statistical significance (*p*-values) and effect sizes calculated using Hedges’ g. Values of 0.20 indicate small effects; 0.50, medium effects; and 0.80, large effects. Furthermore, the Reliable Change Index (RCI) (Jacobson & Truax, 1991) was performed to measure the intra-subject clinical change. The RCI was created to evaluate the effectiveness of a particular treatment, providing information about treatment effects for each subject. It allows a researcher to understand whether the changes observed in each participant are in fact genuine or just due to measurement errors. For the interpretation of the RCI in this study, individuals who achieved scores exceeding 0.84 were allocated to the “global improvement” (GI) group, those scoring below −0.84 were placed in the “global deterioration” (GD) group, and those with scores between these values were assigned to the “no change” (NC) category (Brazão et al., 2015). In this study, differences between groups post-test in the distribution of clinical change categories were examined using chi-square analyses with Fisher’s exact tests, adopting a significance level of 0.05. The strength of the associations between group membership and clinical change categories was estimated using Cramer’s V. Values were interpreted as follows: below 0.10 = negligible association; 0.10 to <0.20 = weak association; 0.20 to <0.40 = moderate association; 0.40 to <0.60 = relatively strong association; 0.60 to <0.80 = strong association; and 0.80 to <1.00 = very strong association.

## 3. Results

### 3.1. Baseline Differences

Baseline comparisons were conducted across all outcome measures. Significant differences between groups emerged only for the negative attributions of CSA within the victim empathy scale (U = 17.50, *p* = 0.43), with the treatment group showing lower levels of cognitive distortions regarding the victim’s experience.

### 3.2. Within-Group and Between-Group Changes

The results revealed statistically significant differences in the expected direction in the treatment condition from pre- to post-test regarding beliefs related to sexuality and CSA, empathy toward victims, and in some interpersonal problems (cold/distant; socially inhibited; nonassertive; overly accommodating; self-sacrificing; intrusive/needy). Regarding EMSs, statistically significant differences were found in the expected direction for emotional inhibition, abandonment/instability, and subjugation. In contrast, when comparing pre- and post-test results in the control condition, no statistically significant differences were observed (see Table 2).

When comparing outcomes between conditions at post-intervention, statistically significant differences emerged between the two groups. Specifically, marginally significant differences (*p* < 0.10) were found in beliefs about CSA, with a moderate effect size. Furthermore, statistically significant differences were observed in both dimensions of empathy toward victims, with large effect sizes.

Regarding EMSs, statistically significant differences between groups were found in emotional inhibition, with a large effect size, and marginally significant differences were observed in social isolation, with a medium effect size.

In relation to interpersonal problems, statistically significant differences were found, with medium-to-large effect sizes in almost all dimensions of the instrument (vindictive/self-centered; cold/distant; socially inhibited; nonassertive; overly accommodating; self-sacrificing; intrusive/needy), with participants in the treatment group reporting fewer interpersonal problems.

### 3.3. Clinical Change in Attitudes Supportive of CSA, Victim Empathy, EMSs and Interpersonal Problems After INSIGHT Program

#### 3.3.1. Attitudes Supportive of CSA and Victim Empathy

Data relating to individual clinical change (RCI) in attitudes supportive of CSA and victim empathy in both groups are presented in Table 3. Results showed significant between-group differences in the distribution of clinical change categories for VES F1 and VES F2, although the results for VES F1 were only marginally significant. Specifically, a higher proportion of participants in the treatment group showed improvement in both VES F1 and VES F2, whereas a higher proportion of participants in the control group were classified in the deterioration or no-change categories. The between-group difference for VES F2 was associated with a large effect size, while the effect size for VES F1 was moderate. No significant between-group differences were found for attitudes supportive of CSA (see Table 3).

#### 3.3.2. EMSs and Interpersonal Problems

Results showed no significant between-group differences in the distribution of clinical change categories for EMSs (see Table 4). Between-group differences emerged in the distribution of clinical change categories for the socially inhibited, non-assertive, overly accommodating, and self-sacrificing dimensions, with a marginally significant difference also observed for the cold/distant dimension. Participants in the treatment group were more frequently classified as improved in these interpersonal problem areas, whereas participants in the control group were more often classified as showing no change or deterioration. These between-group differences were generally accompanied by moderate effect sizes.

## 4. Discussion

The current study aimed to examine the short-term effects of the INSIGHT Program in reducing selected risk factors for sexual recidivism among individuals who victimized girls, namely, EMSs, interpersonal problems, beliefs supportive of CSA, and victim empathy deficits.

The findings indicate that the INSIGHT Program leads to significant improvements in empathy toward victims, with large effect sizes. Specifically, the RCI revealed that a greater proportion of participants in the INSIGHT Program demonstrated meaningful improvements in empathy toward victims, whereas participants in the control group were more frequently classified as showing no change or deterioration. These results corroborate the initial application of the INSIGHT Program in a sample that victimized both girls and boys (Sousa et al., 2026), aligning with one of the intervention’s overall aims. Nevertheless, the results regarding offense-supportive beliefs show that only marginally significant differences emerge between groups in one of the dimensions (beliefs about CSA), and a more detailed analysis at the level of clinical change did not reveal any differences. In fact, although a minority of participants worsened after the INSIGHT Program—namely, one participant—many remain unchanged. Specifically, we found that beliefs supporting CSA do not change and are already at a low level; however, the same does not hold true for sexual preoccupation. These findings may also reflect greater awareness of these thoughts. Thus, it is essential that the program be revised to target these dimensions more intensively, as they are central factors in recidivism (Seto et al., 2023). Taken together, these results suggest that offense-supportive beliefs and victim empathy are distinct constructs, as they respond differently to the program.

The results reveal statistically significant differences between groups in EMS emotional inhibition, with a large effect size, and marginally significant differences in social isolation, with a medium effect size. However, a more detailed analysis of clinical change showed no significant differences in the distribution of clinical change categories for EMSs. Given that one of the program’s aims is to reduce the activation of EMSs, these findings warrant particular attention, especially since a qualitative study with the first participants of the INSIGHT Program indicated that addressing EMSs was central for the positive results (Sousa et al., 2025b). The differences between groups may be due to several factors. First, as noted in previous studies, an increase in schema activation at the later stages of the intervention may reflect greater awareness among participants, as they are often in experiential avoidance at baseline and therefore report lower scores (Brazão et al., 2015). On the other hand, the strategies used throughout the program may not be sufficient to weaken these schemas. Specifically, the use of experiential techniques is challenging, particularly with highly resistant participants, such as individuals who have committed crimes. Research has shown that various internal factors (e.g., shame) and external factors (e.g., noise) can hinder participants’ engagement in these types of exercises, as well as their ability to connect imagined experiences with current problems (de Klerk et al., 2017; Josek et al., 2023). These aspects—especially noise, which is common in prison settings, and shame—may have impacted the work on EMSs. Nonetheless, the results highlight the importance of strengthening the INSIGHT program’s component focused on EMSs, either by allocating more time to it and/or by incorporating alternative methods. As such, some intervention programs have begun to use extended reality in these domains to activate processes that are difficult to access in traditional therapeutic settings (Heikkilä et al., 2024). Extended reality provides an opportunity to gradually expose patients to sources of discomfort or their contextual triggers, allowing them to confront distressing or fear-inducing situations within a controlled environment (Pons et al., 2022).

The importance of improving EMSs is also underscored by the results on interpersonal problems. In fact, the INSIGHT Program proved capable of producing positive changes with medium to strong effect sizes, namely in reducing vindictive/self-Centered, cold/distant, socially inhibited, nonassertive, overly accommodating, self-sacrificing, and intrusive/needy problems. Moreover, between-group differences emerged in the distribution of clinical change categories for the socially inhibited, nonassertive, overly accommodating, and self-sacrificing dimensions, with a marginally significant difference also observed for the sold/distant dimension. These results are highly positive, as interpersonal problems are a central risk factor for recidivism in sexual offending (Seto et al., 2023) and the INSIGHT Program had a strong component on developing interpersonal skills. However, work on EMSs may further enhance these outcomes—particularly in the long term—by promoting more enduring changes, given that EMSs are closely associated with interpersonal competencies (Janovsky et al., 2023).

Bearing in mind that these are only preliminary findings, there are some limitations that should be acknowledged. Specifically, and most importantly, the small sample, the lack of randomization and the lack of follow-up assessment. Randomized control trials are the gold standard for evaluating treatment effectiveness; however, the absence of randomization is highly relevant in community and prison settings, given the associated ethical concerns—namely, the possibility of withholding treatment, which may place potential victims at risk (Tyler et al., 2021; Gouveia et al., 2026). Thus, future studies should aim to include a larger sample. In addition, following the guidelines of the Collaborative Outcome Data Committee (Beech et al., 2007a, 2007b), further measures of program effectiveness should be incorporated, particularly regarding recidivism rates.

Nonetheless, the findings offer important practical and scientific contributions. First, the results—considering both statistical significance and effect sizes—suggest that the INSIGHT Program may be a viable intervention for men who have sexually victimized girls, contributing to reductions in interpersonal problems and increases in victim empathy. This intervention may be implemented in both prison and community settings, provided that inclusion and exclusion criteria are carefully observed. Second, new approaches should be incorporated—such as the use of extended reality—to facilitate work on EMSs by promoting greater in-session activation of these schemas, thereby enhancing therapeutic processing, as well as by allocating more intervention time to this population. Third, research efforts aimed at validating the program’s effectiveness should continue, seeking to include additional outcome measures (e.g., behavioral indicators such as recidivism rates) and a waiting-list control group in order to minimize ethical concerns.

## Figures and Tables

**Table 1:** Characteristics of the participants.

	Participants	
	M	SD
**Age**	45.11	9.04
**Sentence time**	108.53	45.09
**Number of victims**	1.79	1.03
	***n***	**%**
**Setting**		
Prison	17	89.5
Community	2	10.5
**Marital status**		
Single	4	21.1
Married	6	31.6
Divorced/Separated	9	47.4
**Nationality**		
Portuguese	18	94.7
Non-Portuguese	1	5.3
**Educational level**		
4th grade	3	15.8
6th grade	4	21.1
9th grade	9	47.4
12th grade	2	10.5
More than 12th grade	1	5.3
**Previous convictions**	5	26.3
**Previous convictions of CSA**	0	0
**Relationships with victim (s)**		
Family	15	78.9
Acquaintance	3	15.8
Unknown	1	5.3

**Table 2:** Differences in attitudes supportive of CSA, victim empathy, psychopathology and EMSs.

	Treatment Condition					Control Condition					Time × Condition	
	Pre-Test		Post-Test		Z	Pre-Test		Post-Test		Z	*U*	Hedges’ g
	M	SD	M	SD		M	SD	M	SD			
**HSAQ**												
Fator 1	18.20	4.24	14.11	5.09	−1.973 *	18.89	2.67	16.56	5.43	−1.192	28.00	0.26
Fator 2	6.80	2.04	4.80	1.14	−2.388 *	8.00	1.59	7.22	2.77	−0.689	21.50 +	0.47
**VES**												
Fator 1	27.70	14.57	3.60	2.41	−2.803 *	19.63	9.29	15.50	10.13	−1.014	9.000 *	0.64
Fator 2	17.80	4.10	26.60	3.75	−2.670 *	22.75	4.83	22.88	2.53	−0.169	15.50 *	0.50
**Schemas**												
Emotional Deprivation	10.50	6.75	6.30	1.57	−1.602	9.89	5.49	7.67	3.08	−1.753	35.50	0.19
Social Isolation/Alienation	9.90	6.85	6.00	1.63	−1.472	9.89	5.49	9.44	4.19	−0.845	23.00 +	0.44
Emotional Inhibition	11.60	5.82	7.00	3.53	−2.375 *	11.89	4.81	10.89	3.14	−0.946	18.00 *	0.52
Defectiveness/Shame	9.00	6.43	6.30	1.89	−1.334	8.11	3.72	7.25	2.77	−1.187	33.00	0.16
Mistrust/Abuse	13.00	6.43	9.40	3.40	−1.423	11.22	2.82	10.44	5.56	−0.424	38.00	0.14
Abandonment/Instability	12.22	3.93	9.00	4.00	−2.254 *	13.75	5.65	10.77	5.56	−1.701	37.50	0.13
Subjugation	10.60	5.58	6.70	2.63	−1.976 *	8.56	2.51	6.89	2.03	−1.827	44.00	0.02
**Interpersonal problems**												
Domineering/controlling	1.44	2.92	0.10	0.32	−1.841 +	0.78	1.39	0.67	1.00	−0.378	33.00	0.32
Vindictive/self-centered	3.67	3.43	0.90	0.99	−1.951 +	7.00	3.60	5.63	3.85	−1.199	11.00 *	0.60
Cold/distant	5.00	3.43	0.20	0.63	−2.524 *	8.00	5.31	7.00	4.87	−1.035	11.00 *	0.71
Socially inhibited	5.22	3.03	0.70	1.16	−2.673 *	5.78	3.35	5.22	3.70	−0.351	13.00 *	0.64
Nonassertive	7.22	3.42	1.10	0.74	−2.675 *	7.38	3.29	6.78	5.09	0.000	10.50 *	0.66
Overly accommodating	4.50	2.37	1.10	0.99	−2.814 *	5.63	3.02	4.89	2.67	−1.018	7.00 ***	0.72
Self-sacrificing	6.80	3.58	4.50	4.58	−2.203 *	6.71	3.15	7.67	4.33	−1.084	22.00 +	0.43
Intrusive/needy	4.30	3.80	2.00	2.45	−2.375 *	5.00	3.66	4.33	2.18	−0.458	19.50 *	0.49

*** *p* < 0.001; * *p* < 0.05; + *p* < 0.10.

**Table 3:** Reliable clinical change for attitudes supportive of CSA and victim empathy.

Variable	Categories	Treatment Condition	Control Condition	Fisher’s	*p*	Cramer’s V
		N	N			
HSAQ F1	GI	1	1	0.835	0.809	0.172
	NC	4	5			
	GD	5	3			
HSAQ F2	GI	0	1	1.423	0.650	0.275
	NC	5	5			
	GD	5	3			
VES F1	GI	0	2	3.082	0.076	0.425
	NC	1	2			
	GD	9	5			
VES F2	GI	9	2	8.664	0.007	0.688
	NC	1	6			
	GD	0	1			

Note. GI = Global improvement; NC = no change; GD = global deterioration; HSAQ F1 = misconceptions about child sexual abuse; HSAQ F2 = sexual drive/preoccupation; VES F1 = positive misattributions of pleasure in sexual abuse; VES F2 = negative attributions regarding CSA.

**Table 4:** Reliable clinical change for EMSs and interpersonal problems.

Variable	Categories	Treatment Condition	Control Condition	Fisher’s	*p*	Cramer’s V
		N	N			
**Schemas**						
Emotional Deprivation	GI	5	5	0.474	1.00	0.122
	NC	3	3			
	GD	2	1			
Social Isolation/Alienation	GI	5	5	2.565	0.424	0.381
	NC	4	1			
	GD	1	3			
Emotional Inhibition	GI	7	4	1.861	0.475	0.317
	NC	3	4			
	GD	0	1			
Defectiveness/Shame	GI	4	4	0.311	1.00	0.069
	NC	4	3			
	GD	2	2			
Mistrust/Abuse	GI	5	5	1.179	0.70	0.246
	NC	3	1			
	GD	2	3	0.786	0.800	0.159
Abandonment/Instability	GI	7	5			
	NC	2	3			
	GD	1	1			
Subjugation	GI	7	5	1.625	0.546	0.292
	NC	1	3			
	GD	2	1			
**Interpersonal problems**						
Domineering/controlling	GI	4	2	2.344	0.443	0.378
	NC	6	5			
	GD	0	2			
Vindictive/self-centered	GI	7	6	0.862	1.000	0.191
	NC	2	1			
	GD	1	2			
Cold/distant	GI	8	4	3.112	0.071	0.429
	NC	2	3			
	GD	0	2			
Socially inhibited	GI	10	5	5.093	0.033	0.544
	NC	0	1			
	GD	0	3			
Non-assertive	GI	10	5	5.093	0.033	0.544
	NC	0	1			
	GD	0	3			
Overly accommodating	GI	10	5	5.093	0.033	0.544
	NC	0	1			
	GD	0	3			
Self-sacrificing	GI	8	2	6.327	0.030	0.588
	NC	1	2			
	GD	1	5			
Intrusive/needy	GI	7	5	2.172	0.528	0.362
	NC	3	2			
	GD	0	2			

Note. GI = Global improvement; NC = no change; GD = global deterioration.

## Data Availability

The data that support the findings of this study are available on request from the corresponding author. The data is not publicly available due to their containing information that could compromise the privacy of research participants.
